# Circulating platelet-neutrophil aggregates characterize the development of type 1 diabetes in humans and NOD mice

**DOI:** 10.1172/jci.insight.153993

**Published:** 2022-01-25

**Authors:** Sarah K. Popp, Federica Vecchio, Debra J. Brown, Riho Fukuda, Yuri Suzuki, Yuma Takeda, Rikako Wakamatsu, Mahalakshmi A. Sarma, Jessica Garrett, Anna Giovenzana, Emanuele Bosi, Antony R.A. Lafferty, Karen J. Brown, Elizabeth E. Gardiner, Lucy A. Coupland, Helen E. Thomas, Beng H. Chong, Christopher R. Parish, Manuela Battaglia, Alessandra Petrelli, Charmaine J. Simeonovic

**Affiliations:** 1 Department of Immunology and Infectious Disease, The John Curtin School of Medical Research, The Australian National University (ANU), Canberra, Australia.; 2 San Raffaele Diabetes Research Institute, IRCCS Ospedale San Raffaele, Milan, Italy.; 3 Tokyo Medical and Dental University, Bunkyo City, Tokyo, Japan.; 4 ACRF Department of Cancer Biology and Therapeutics, The John Curtin School of Medical Research, ANU, Canberra, Australia.; 5 San Raffaele Vita Salute University, Milan, Italy.; 6 Department of Pediatrics, The Canberra Hospital, Canberra, Australia.; 7 St. Vincent’s Institute of Medical Research, Melbourne, Australia.; 8 Hematology Research Unit, St. George and Sutherland Clinical School, University of New South Wales, Sydney, Australia.

**Keywords:** Autoimmunity, Diabetes, Neutrophils, Platelets

## Abstract

Platelet-neutrophil aggregates (PNAs) facilitate neutrophil activation and migration and could underpin the recruitment of neutrophils to the pancreas during type 1 diabetes (T1D) pathogenesis. PNAs, measured by flow cytometry, were significantly elevated in the circulation of autoantibody-positive (Aab^+^) children and new-onset T1D children, as well as in pre-T1D (at 4 weeks and 10–12 weeks) and T1D-onset NOD mice, compared with relevant controls, and PNAs were characterized by activated P-selectin^+^ platelets. PNAs were similarly increased in pre-T1D and T1D-onset NOD isolated islets/insulitis, and immunofluorescence staining revealed increased islet-associated neutrophil extracellular trap (NET) products (myeloperoxidase [MPO] and citrullinated histones [CitH3]) in NOD pancreata. In vitro, cell-free histones and NETs induced islet cell damage, which was prevented by the small polyanionic drug methyl cellobiose sulfate (mCBS) that binds to histones and neutralizes their pathological effects. Elevated circulating PNAs could, therefore, act as an innate immune and pathogenic biomarker of T1D autoimmunity. Platelet hyperreactivity within PNAs appears to represent a previously unrecognized hematological abnormality that precedes T1D onset. In summary, PNAs could contribute to the pathogenesis of T1D and potentially function as a pre-T1D diagnostic.

## Graphical abstract

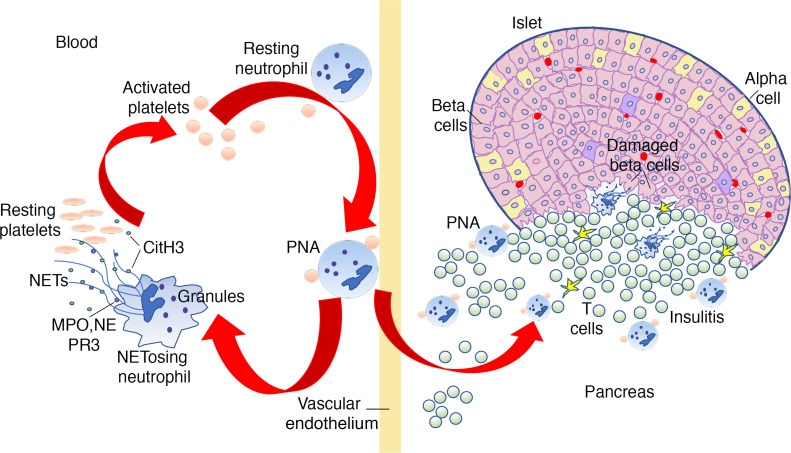

## Introduction

Type 1 diabetes (T1D) is an autoimmune disease that selectively destroys the insulin-producing β cells within the islets of Langerhans in the pancreas (1–3). Generally, asymptomatic T1D disease progresses slowly, due to both the asynchronous attack of islets and to preferential targeting of different regions of the pancreas during disease progression (4, 5). T lymphocytes have been identified in human insulitis lesions, strongly suggesting a role for T cell–mediated autoimmune damage of β cells (6, 7). In support, T cell–targeted intervention therapy (non-Fc receptor binding anti-CD3 mAb or teplizumab) in pre-T1D individuals delays the clinical onset of T1D (8). However, T cells from T1D donors have displayed considerable heterogeneity in response to conventional β cell antigens in vitro, with some donors being identified as nonresponders (9). Such findings raise the possibility that other leukocyte populations may actively contribute to β cell destruction.

Recent reports have revealed a role for the innate immune system in T1D development. Circulating neutrophils decline in children with recent-onset T1D and in autoantibody^+^ (Aab^+^) individuals with an increased risk of developing T1D (defined as “pre-T1D” children) (10–13). Conversely, neutrophil extracellular traps (NETs), which are composed of a complex network of neutrophil-derived chromatin (i.e., DNA and histones) decorated with enzymes derived from neutrophil granules, such as neutrophil elastase (NE) and proteinase 3 (PR3), were increased in the circulation of young individuals after T1D onset. The catalytic activity of NE and PR3 in peripheral blood was also elevated and accompanied by an increased number of islet Aabs. Collectively, these findings provided strong evidence linking T1D onset and neutrophil activation (13). Although the precise contribution of neutrophils to human T1D disease development is unclear, the detection of neutrophils and NET products in Aab^+^ and T1D pancreas sections suggest that activated neutrophils may have a pathogenic role (12, 14).

Studies of the NOD mouse model of spontaneous autoimmune diabetes have provided additional support for a role for neutrophils in the pathogenesis of T1D. Young 2–3 week old NOD mice showed increased islet-associated neutrophils with evidence of neutrophil activation and NET release (15). Compared with non-T1D progressors, NOD mice that progressed to T1D-onset demonstrated increased activity of circulating NE and PR3 from 4 weeks of age and throughout pre-T1D disease progression (13). Furthermore, transient depletion of Ly6C^+^ myeloid cells (including Ly6G^+^ neutrophils) in NOD neonates significantly reduced the incidence of T1D in adulthood (15). Together, these findings support the notion that neutrophils participate in the initiation and progression of T1D autoimmunity.

How neutrophils are activated to release NETs in T1D is not understood. In Aab-mediated autoimmune diseases such as systemic lupus erythematosis, small vessel vasculitis, and rheumatoid arthritis, platelets contribute to neutrophil activation (16–19). Activated platelets engage neutrophils via interactions between platelet cell surface CD62P (P-selectin) and neutrophil P-selectin binding glycoprotein ligand 1 (PSGL1) (20–23), soluble High Mobility Group Box protein 1 (HMGB1; platelet-derived) (24, 25) and neutrophil Receptor for Advanced Glycation End-products (RAGE), or platelet glycoprotein (GP) Ibα and neutrophil αMβ2 (Mac-1) (26–28), with these interactions triggering the release of NETs (24). However, platelets are also activated by neutrophil NETs/histones and the serine proteases Cathepsin G and NE (20, 21, 29–33), creating a continuous cycle of platelet-neutrophil activation (21). Such interactions can result in hyperactive platelets and increased levels of platelet-leukocyte aggregates in the circulation, which have been reported to be associated with secondary vascular complications of T1D (34–37). Specifically, histones activate platelets by binding to cell surface toll-like receptor 4 (TLR4) or TLR2, promoting the formation of platelet-neutrophil aggregates (PNAs) (32, 38). Importantly, the interaction between platelet P-selectin and neutrophil PSGL1 has been shown to play an important role in neutrophil extravasation and trafficking to tissues (22, 39). Raised circulating levels of platelet-derived proteins correlate with recent-onset T1D, including circulating P-selectin (a platelet activation marker) and proplatelet basic protein (PPBP), which is cleaved to produce CXCL7, a chemokine that primes neutrophil migration into tissues (40–43). Despite this knowledge, the mechanisms promoting neutrophil activation, mobilization, and recruitment to the pancreas in T1D, as well as the susceptibility of islets to NET-induced damage, remain undefined.

In this study, we investigated a role for platelets in the activation of neutrophils during the development of T1D in humans and NOD mice by examining platelet-neutrophil interactions, identified by the formation of PNAs. Our findings suggest that PNAs are elevated both in the circulation and the islets/insulitis of NOD mice, and they are also increased in the peripheral blood of human T1D during the disease development. Thus, PNAs possibly represent a pathogenic and innate immune biomarker of asymptomatic T1D autoimmunity. In addition, we reveal platelet hyperreactivity in PNAs as a previously unrecognized hematological abnormality that precedes T1D onset, potentially contributing to T1D disease progression by promoting neutrophil activation and recruitment to the pancreas.

## Results

### PNAs increase in peripheral blood during T1D development in NOD mice.

**Figure 1:**
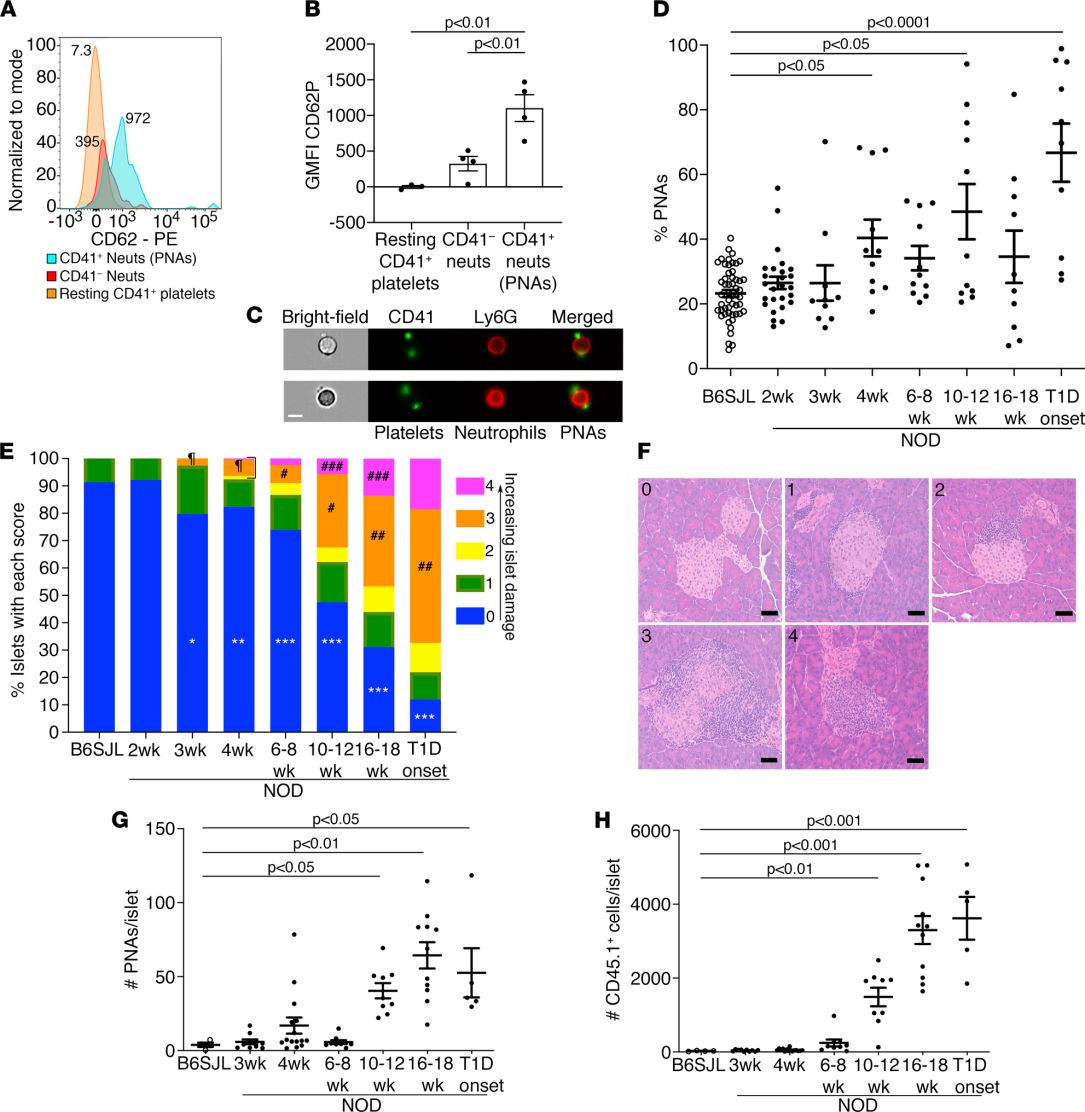
PNA levels are elevated in NOD blood and islets during T1D development. (**A**) Representative histograms show CD62P geometric MFI (geometric mean fluorescence intensity) on resting platelets, CD41^–^ neutrophils (Neuts), and CD41^+^ neutrophils (PNAs) in 10- to 12-week NOD blood. (**B**) Mean CD62P GMFI ± SEM for 10- to 12-week NOD resting platelets, CD41^–^ neutrophils, and PNAs. *n* = 3–4 mice/group. Ordinary 1-way ANOVA with Tukey’s multiple comparisons test was used. (**C**) ImageStream flow cytometry images show bight-field, CD41^+^ platelets (green), Ly6G^+^ neutrophils (red), and platelets aggregated with neutrophils (PNAs; merged images) in pre-T1D NOD blood. Scale bar: 7 μm. (**D**) Circulating PNAs in NOD females at 2–18 weeks of age and at T1D onset. Data show mean ± SEM, *n* = 10–26/NOD group; *n* = 52 B6SJL adult females/group; nonparametric Kruskal-Wallis test and Dunn’s multiple comparisons test. (**E**) Histological assessment of host pancreata shows the percentage of islets with each score (scores 0–4), *n* = 154–300 islets examined/group; *n* = 10–25 pancreata/group except for 2 weeks, where *n* = 4 pancreata/group. Statistical analyses compared the percentage of islets with a histological score between NOD and B6SJL groups or between NOD groups at different ages (including T1D onset). *P = 0.0019, **P = 0.0100, ***P < 0.0001, compared with B6SJL controls; ^¶^*P* = 0.0209, 3-week versus 4-week NOD; ^#^*P* < 0.0001, 6- to 8-week versus 10- to 12-week NOD; ^##^*P* = 0.0004, 16- to 18-week versus T1D-onset NOD; ^###^*P* = 0.0031, 10- to 12-week versus 16- to 18-week NOD; Fisher’s exact test. (**F**) Representative islet images showing histological scoring criteria: 0, intact islets without insulitis/inflammation; 1, little to mild periislet accumulation of leukocytes; 2, pronounced nondestructive insulitis; 3, destructive insulitis with islet-infiltrating leukocytes; 4, complete islet destruction with little or no islet tissue remaining. Scale bar: 50 μm. (**G** and **H**) Flow cytometry analysis of PNAs/isolated islet for NOD females at 3–18 weeks and T1D onset and corresponding CD45^+^ leukocytes/islet. Data show mean ± SEM; NOD/Lt, *n* = 5–15 donors/group; B6SJL, *n* = 12 donors/4 experiments/group; nonparametric Kruskal-Wallis test and Dunn’s multiple comparisons test.

PNAs were measured in whole blood by flow cytometry (Supplemental Figure 1; supplemental material available online with this article; https://doi.org/10.1172/jci.insight.153993DS1). In PNAs of adult NOD female mice, CD62P expression, a marker of platelet activation (44), was > 1000-fold higher than for resting platelets (*P* < 0.01) and 3.4-fold higher than for CD41^–^ neutrophils (*P* < 0.01) (Figure 1, A and B), dismissing the possibility that the platelets inadvertently adhered to neutrophils during in vitro manipulation. The unusual expression of CD62P on CD41^–^ neutrophils was most likely due to the binding of soluble CD62P, which can be released from activated platelets (45). ImageStream multispectral flow cytometry visually confirmed PNAs in T1D-onset and pre-T1D NOD mouse blood (Figure 1C). Cross-sectional analysis of PNAs in NOD females at 2, 3, 4, 6–8, 10–12, and 16–18 weeks of age, as well as at T1D onset (day 0 [d0] to d1 after onset) revealed that, unlike B6SJL controls, blood PNA levels in adult NOD mice showed striking variability and an apparent bimodal distribution. Compared with PNA levels in control nonautoimmune B6SJL mice (23.2% ± 1.1%), mean circulating NOD PNAs were significantly elevated 1.7-fold to 40.4% ± 5.7% at 4 weeks of age, 2.1-fold to 48.6% ± 8.5% at 10–12 weeks, and 2.9-fold to 66.8% ± 9.0% at T1D onset (Figure 1D). When the histology of the host pancreata from Figure 1D was analyzed in a blinded fashion, the increased circulating PNAs at 4 weeks of age were found to be associated with increased islet inflammation, including initial signs of destructive insulitis (Figure 1, E and F); 7.6% of islets were graded with scores 2–4 (inclusive) at 4 weeks of age versus 2.6% at 3 weeks (*P* = 0.0209). The elevated PNAs at 10–12 weeks correlated with a 4-fold increase in islets infiltrated by insulitis leukocytes, with 26.8% of islets showing destructive insulitis (score 3) compared with 6.7% at 6–8 weeks (*P* < 0.0001). Similarly, at T1D onset, increased PNAs were accompanied by a 1.5-fold increase in the percentage of islets with leukocyte infiltration (score 3), compared with 16–18 weeks (*P* = 0.0004). In parallel, intact islets significantly declined to 82.4%, 47.6%, and 12.0% at 4 weeks, 10–12 weeks, and T1D onset, respectively, compared with nonautoimmune control islets (Figure 1, E and F). Additionally, the spikes in circulating PNA levels in NOD mice did not correlate with increased circulating neutrophils or platelets at the same time points; in fact, T1D onset was associated with a significant ~50% reduction in the neutrophil count compared with levels at 16–18 weeks (Supplemental Figure 2). In summary, circulating PNAs increased during T1D development in NOD mice, with spike PNA levels correlating with the degree of insulitis.

### PNA levels in NOD islets are elevated during T1D development.

NOD islet–associated PNA levels showed a transient 4.3-fold increase at 4 weeks of age (16.9 ± 5.4 PNAs/NOD islet versus 3.9 ± 1.4 PNAs/B6SJL control islet) and appeared to be more variable than for islets from donors at 3 weeks and 6–8 weeks (Figure 1G), a finding that accounted for the failure to achieve statistical significance. Islet/insulitis PNAs were more prevalent at 10–12 weeks (40.5 ± 5.2 PNAs/islet), 16–18 weeks (64.4 ± 8.9 PNAs/islet), and T1D onset (52.6 ± 16.7 PNAs/islet), representing a significant 4- to 17-fold increase during disease progression, compared with control nonautoimmune B6SJL islets (3.9 ± 1.4 PNAs/B6SJL islet; Figure 1G). The profile for increased NOD islet–associated PNAs correlated with a 58- to 140-fold increase in insulitis CD45^+^ leukocytes from 10–12 weeks to T1D onset versus background levels in control islets (Figure 1H) and, like blood PNA levels (Figure 1D), with histological evidence for increased islet inflammation/destructive insulitis in the pancreas of separate groups of age-matched NOD mice (Figure 1E). The initial increase in islet PNAs at 4 weeks and subsequent gross leukocyte infiltration into islets from 10–12 weeks suggest that islet PNAs may contribute both to mild β cell damage early during the initiation of autoimmunity in young NOD mice and as components of the destructive insulitis responsible for the autoimmune destruction of β cells in adult NOD mice.

### Products of activated neutrophils are detected in NOD pancreas.

**Figure 2:**
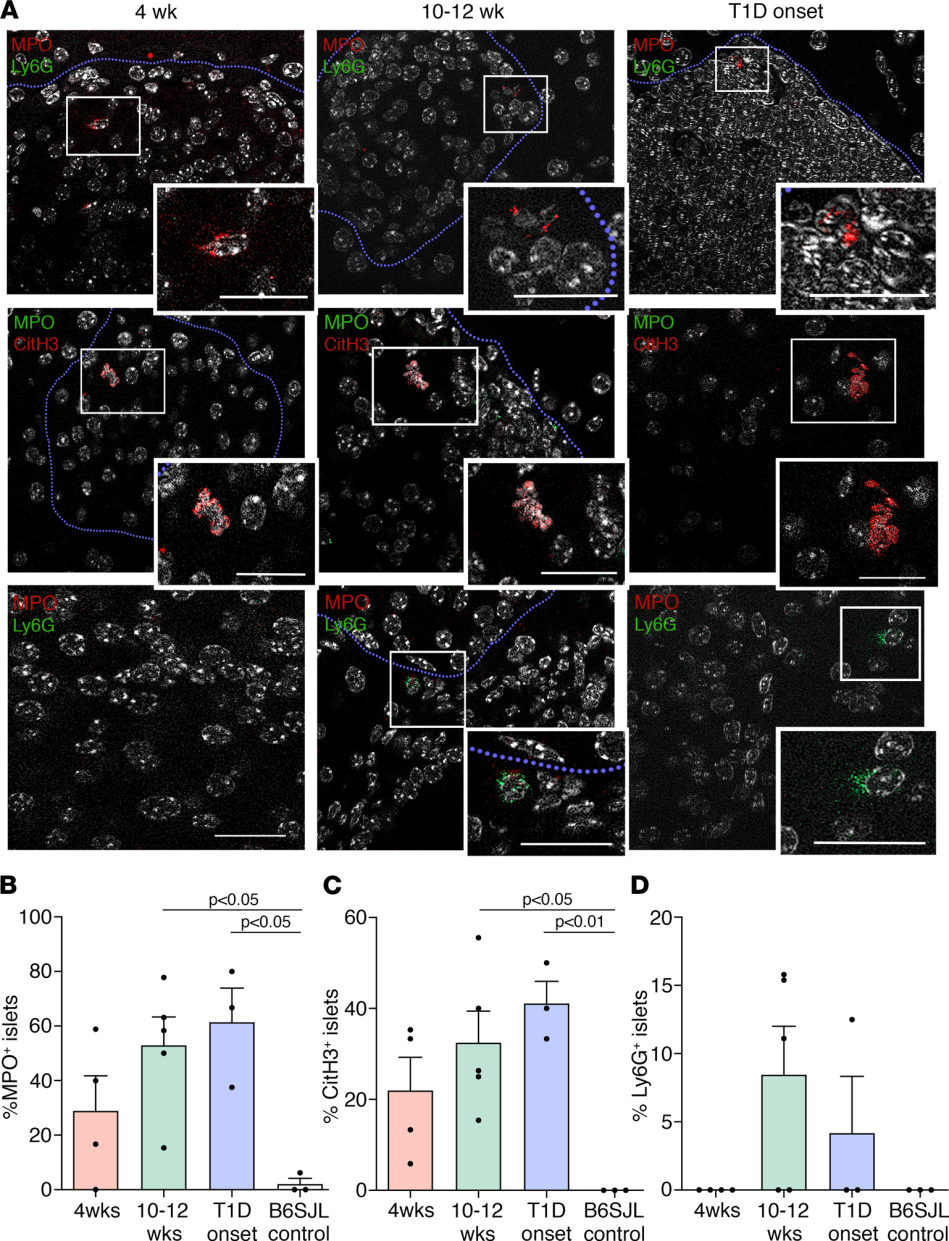
NETs and NET products are localized in NOD pancreas. (**A**) Representative images show immunolocalization of MPO, CitH3, and Ly6G in NOD pancreas sections. Blue dashed lines define an islet border. DAPI (white) identifies DNA. Scale bar: 20 μm. (**B**–**D**) Frequency of MPO^+^, CitH3^+^, and Ly6G^+^ islets in NOD pancreata during T1D development as the percentage of total islets. In total, 1–2 pancreas sections were stained per sample. B6SJL islets were examined as nonautoimmune controls. Data show mean ± SEM; *n* = 3–5 pancreata/group; *n* = 19–63 islets examined/group. One-way ANOVA, Tukey’s multiple comparisons test.

We next investigated whether islet-associated PNAs correlated with the detection of neutrophils and NET products (evidence of neutrophil activation) in NOD pancreas by immunofluorescence staining. NOD pancreata at 10–12 weeks and T1D onset showed a significant 25- to 29-fold higher frequency of MPO^+^ islets (52.9% ± 10.4% and 61.4% ± 12.6%, respectively) than control B6SJL islets (2.1% ± 2.1%; Figure 2, A and B); in parallel, 32.5% ± 7.0% (*P* < 0.05) and 41.1% ± 4.8% (*P* < 0.01) of islets, respectively, were positive for CitH3^+^, compared with B6SJL islets in which CitH3 was undetectable (Figure 2C). In 4-week NOD pancreas, the relative frequency of MPO^+^ islets (28.9% ± 12.9%) and CitH3^+^ islets (22.0% ± 7.3%) was substantially higher than in normal B6SJL pancreas (2.1% ± 2.1% and 0%, respectively), but these differences were not statistically significant (Figure 2, B and C). Overall, staining for the neutrophil-derived products CitH3 and MPO within islets was more readily detected than for neutrophils identified by the Ly6G cell surface marker; the percentage of Ly6G^+^ islets was 8.5% ± 3.5% and 4.2% ± 4.2% for 10–12 weeks and T1D onset, respectively (Figure 2D). Islet-associated NETs were rarely found, and Ly6G^+^ neutrophils appeared to be more frequently observed in peri-islet regions rather than inside islets (Supplemental Figure 3 compared with Figure 2D). Overall, the frequency of NOD islets showing MPO^+^ and CitH3^+^ staining, representing markers of activated neutrophils, increased during the progression of T1D disease.

### Histones/NETs induce mouse and human islet/β cell damage.

**Figure 3:**
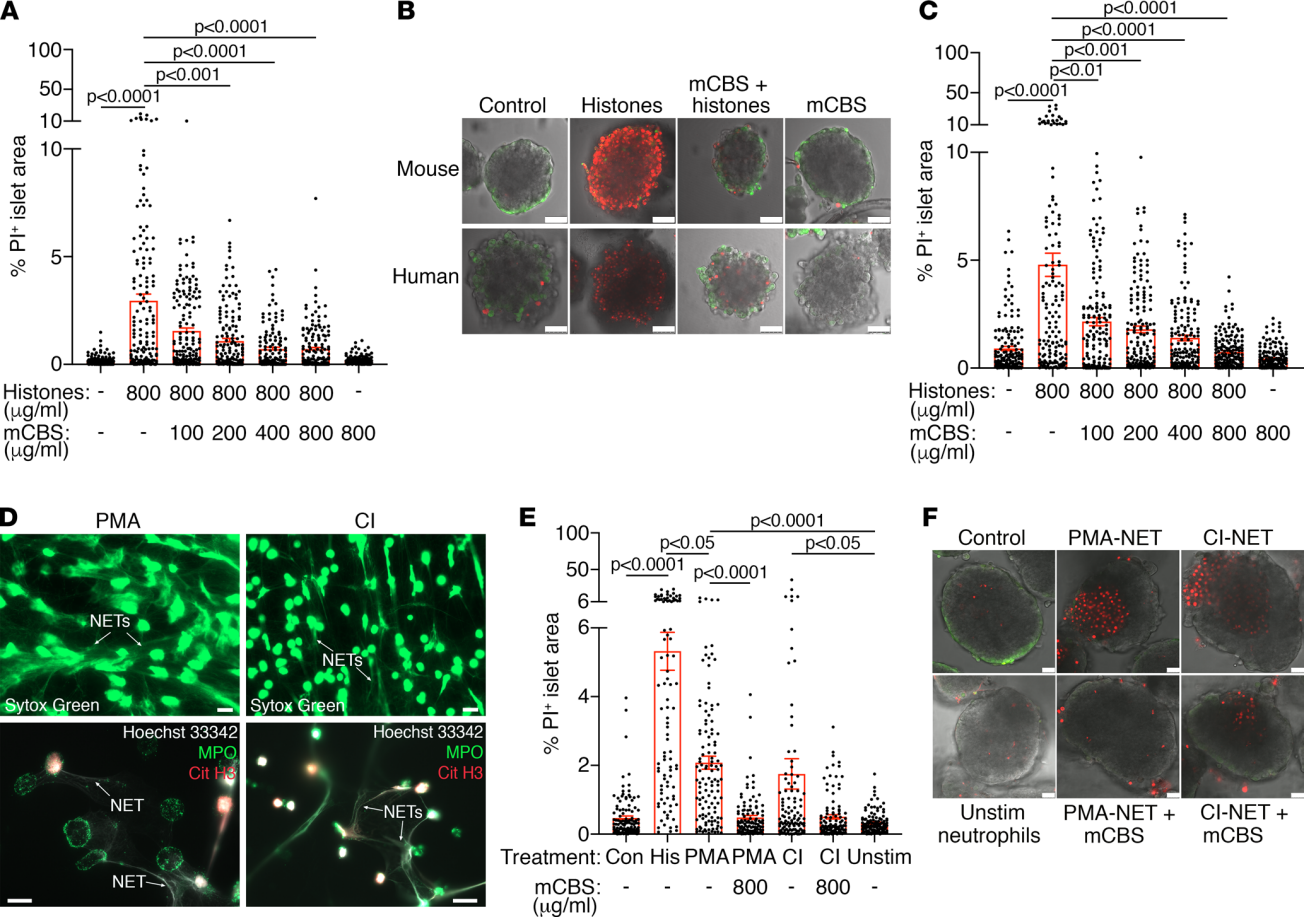
Histone- and NET-induced damage of islets is prevented by mCBS. (**A**) BALB/c mouse islets treated for 2 hours with 800 μg/mL histones and labeled with PI to identify islet cell death (% PI^+^ islet area) by confocal microscopy and morphometry. Data show mean ± SEM for islets examined in each group. *n* = 3 experiments/group, *n* = 24–30 islets analyzed/group, *n* = 4–7 focal planes at 30 μm intervals examined from a *Z* stack for each islet, acquired by confocal microscopy. Nonparametric Kruskal-Wallis test with Dunn’s multiple comparisons test. (**B**) Confocal images of mouse (top panels) and human (bottom panels) islets labeled with PI (red) to identify damaged/dead islet cells and Calcein-AM (green) to identify viable cells. Scale bar: 50 μm. (**C**) Human islets treated for 18 hours with 800 μg/mL histones + 100–800 μg/mL mCBS. Data show mean percentage of PI^+^ islet area ± SEM for islets in each group; *n* = 3 experiments, *n* = 30 islets/group, *n* = 4–7 focal planes were examined, as in **A**. Nonparametric Kruskal-Wallis test with Dunn’s multiple comparisons test. (**D**) Fluorescence microscopy of PMA- and CI-treated human neutrophils demonstrate release of NETs labeled with SYTOX Green (top panels); merged images confirm that NETs consisted of extracellular DNA (Hoechst 33342; white), MPO (green), and CitH3 (red). Scale bar: 20 μm. (**E**) Isolated mouse islets cultured with PMA-NETs, CI-NETs, or histones (His) for 17 hours. Data show mean percentage of PI^+^ islet area ± SEM; *n* = 3 experiments, *n* = 28–30 islets analyzed/group, *n* = 3–5 focal planes were examined as in **A**. Nonparametric Kruskal-Wallis test with Dunn’s multiple comparisons test. (**F**) Confocal images of isolated mouse islets treated with PMA-NETs and CI-NETs for 17 hours and labeled with PI (red) and Calcein-AM (green). Scale bar: 25 μm.

The potential for cell-free histones and NETs to damage islets/β cells was evaluated in vitro using commercially available calf thymus histones and NETs generated from human neutrophils stimulated with phorbol myristate acetate (PMA) or calcium ionophore (A23187; CI). A significant 8.5- to 25-fold increase in the death of islet cells was observed after treatment of isolated mouse islets with 100–400 μg/mL of histones for 2–48 hours, compared with corresponding control islets (Supplemental Figure 4, A–C). Treatment of mouse islets with 800 μg/mL of histones for 2 hours induced maximal islet damage, ~20-fold higher than controls (*P* < 0.0001; Supplemental Figure 5 and Figure 3, A and B). Treatment of isolated human islets with 800 μg/mL of histones increased the PI^+^ islet area by 5.2-fold compared with medium-treated control islets (*P* < 0.0001; Figure 3, B and C). Human islets cultured with FITC-labeled histones demonstrated that histones were localized to the islet periphery, consistent with the observed peripheral distribution of PI^+^ islet cells (Supplemental Figure 6, A and B). Thus, the peripheral damage induced by extracellular histones correlated with the limited penetration of the histones into the islet cell mass. We next tested whether NETs, a physiologically relevant source of extracellular histones, damaged isolated mouse islets in vitro. The release of NETs from human neutrophils stimulated with PMA or CI was confirmed by labeling of extracellular DNA with the fluorescent dyes SYTOX Green or Hoechst 33342, as well as by costaining for CitH3 and MPO (Figure 3D). Treatment of mouse islets with PMA-induced NETs (PMA-NETs) and CI-induced NETs (CI-NETs) induced a significant 5- to 6-fold increase in islet cell death, compared with islets treated with unstimulated neutrophils (Figure 3, E and F). In addition, NET-induced islet damage represented 33%–39% of the PI^+^ islet area after treatment with calf thymus histones (Figure 3E). The less severe islet injury induced by NETs was associated with focal damage at the islet periphery (Figure 3F) and was in striking contrast to the more extensive damage induced around the entire islet border by histones (Figure 3B, upper panel). These differences are likely to be partly due to the restricted site of interaction between plastic-bound NETs and the nearby partial surface of the islet versus the greater accessibility for free histones (in suspension) to interact with the entire islet surface. In addition, since there exists a 1:1 ratio of DNA to protein in chromatin (46), the histone content of PMA- and CI-NETs (with DNA content ≤ 1 μg /mL) was much lower than for free calf thymus histones (200–800 μg/mL), resulting in less toxicity for the islets. Nevertheless, this study demonstrated significant NET-induced toxicity for mouse islets in vitro.

### mCBS protects islets from histone and NET-mediated damage.

The small polyanionic drug, methyl cellobiose sulfate (mCBS), which neutralizes the high positive charge of histones, was recently reported to prevent a variety of hematological disorders, including experimental models of sepsis, ischemia reperfusion injury, and deep vein thrombosis, and it was also reported to inhibit histone-induced platelet activation in vitro (47) and platelet-fibrin aggregates (48). We therefore tested whether mCBS could protect islets from histone- and NET-induced damage. In vitro, isolated normal BALB/c mouse islets treated acutely for 2 hours with 800 μg/mL of histones and 200, 400, or 800 μg/mL of mCBS showed a significant reduction in the percentage of PI^+^ islet area to 37%, 24%, and 24% of histone-only–treated controls, respectively. No toxicity was observed when islets were treated with mCBS alone (Figure 3, A and B). Similarly, treatment of human islets with 800 μg/mL of histones and mCBS (100–400 μg/mL) significantly reduced islet cell death to 29.6%–45.5% of histone treatment alone, and 800 μg/mL of mCBS reduced the PI^+^ islet area to 15.8% of histone-only controls (Figure 3, B and C). In parallel, we also found that mCBS (800 μg/mL) reduced PMA-NET– and CI-NET–mediated damage of mouse islets in vitro to 23% of PMA-NET treatment alone (*P* < 0.0001) and 29% of CI-NET–induced damage (Figure 3, E and F). In vivo treatment of young and prediabetic adult NOD mice with mCBS, however, did not significantly alter the incidence of T1D (Supplemental Methods and Supplemental Figure 7, A and B). In summary, histone- and NET-induced damage of islets in vitro was prevented by the small polyanionic drug mCBS.

### Circulating PNAs increase during T1D development in humans.

**Figure 4:**
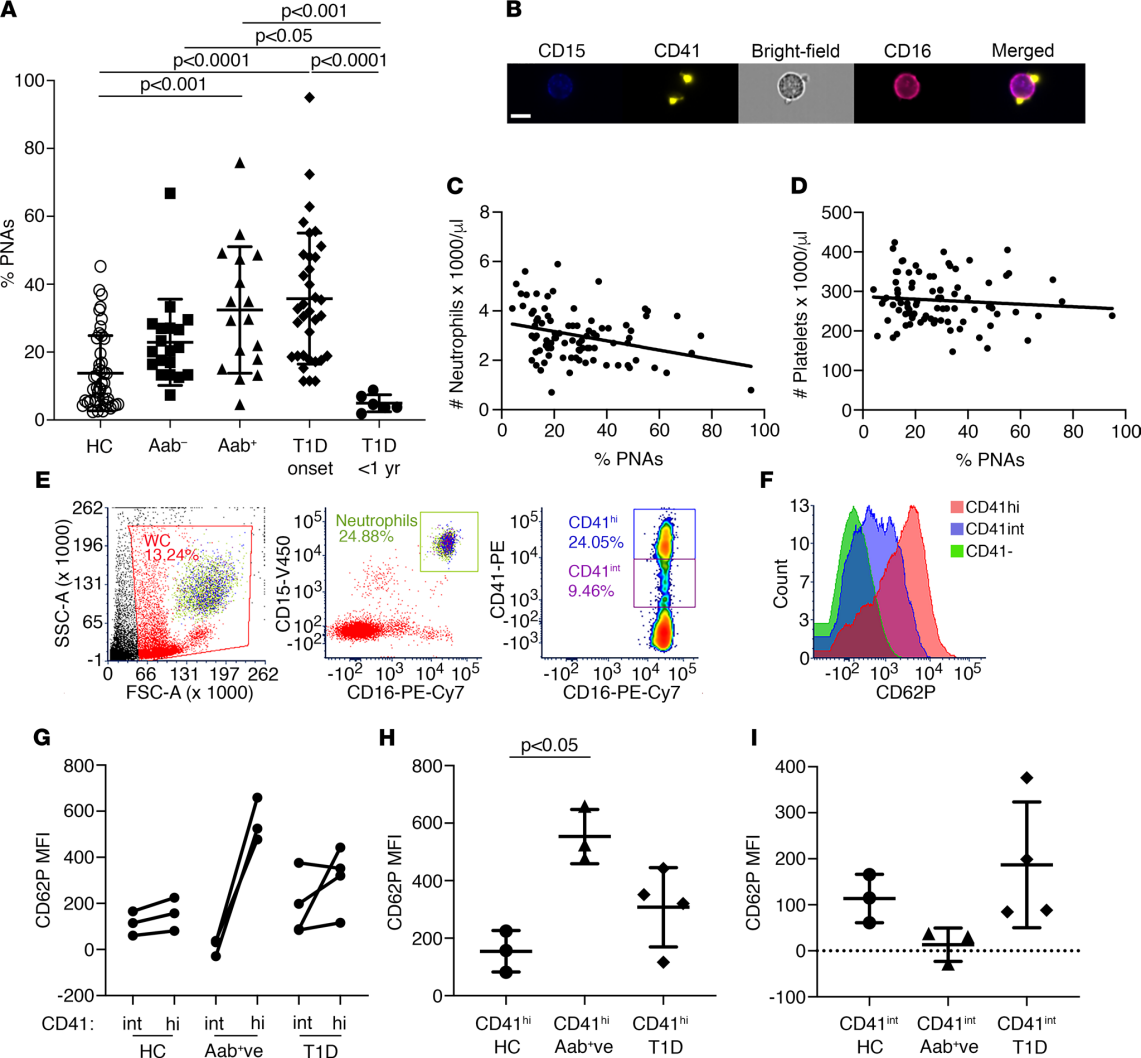
Circulating PNAs increase during T1D development in humans. (**A**) PNAs in whole blood from children aged 5–16 years who were Aab^–^ (*n* = 19), Aab^+^ (*n* = 17; includes repeated measures for *n* = 3 donors; each donor remained Aab^+^ and pre-T1D), recent-onset T1D (~10 days after diagnosis; *n* = 34) and T1D < 1 year (2–11 months after diagnosis; *n* = 6); healthy children (HC; *n* = 46) at 5–16 years of age served as controls. Data show mean ± SD. Nonparametric Kruskal-Wallis test with Dunn’s multiple comparisons test. (**B**) Representative ImageStream flow cytometry identified CD41^+^ platelets (yellow) and CD15^+^ (blue), CD16^+^ (crimson) neutrophils in pre-T1D human blood; merged image shows 2 platelets aggregated with a CD15^+^CD16^+^ (pink) neutrophil (PNA). Scale bar: 7 μm. (**C** and **D**) Correlation analyses show a negative correlation between circulating PNAs and neutrophils from healthy control, Aab^–^, Aab^+^ (including *n* = 3 repeat measures) and T1D-onset groups; Spearman nonparametric correlation test, *P* = 0.0041 (2-tailed), *r* = –0.3035. Linear regression line has been included in the graph to highlight the negative correlation. No correlation was observed between platelet levels and PNAs; Spearman nonparametric correlation test, ns (2-tailed), *r* = –0.1012. (**E**) Gating strategy for flow cytometry analysis of CD41^hi^, CD41^int^ platelet subgroups in PNAs. (**F**) Representative histograms show relative fluorescence intensity for CD62P in CD41^–^ neutrophils (green), CD41^int^ platelets in PNAs (purple), and CD41^hi^ platelets in PNAs (red). (**G**–**I**) MFI of CD62P after gating on CD41^hi^ and CD41^int^ platelets in PNAs in fresh blood from healthy pediatric controls, pre-T1D Aab^+^ children and children at T1D onset (**G**); CD41^hi^ PNAs (**H**), and CD41^int^ PNAs (**I**) show mean CD62P MFI ± SD. (**G**) Wilcoxon test; (**H**–**I**) nonparametric Kruskal Wallis test with Dunn’s multiple comparisons test.

Circulating PNAs were analyzed in whole human blood by flow cytometry (Supplemental Figure 8) for a cross-sectional study of Aab^–^ and Aab^+^ children with a first- or second-degree relative with T1D, children with new-onset T1D (~10 days after clinical T1D diagnosis), T1D pediatric patients < 1 year after T1D onset (2–11 months after T1D diagnosis), and normal healthy children, within the age range 5–16 years (see Supplemental Table 1 for summary of demographics). We demonstrated a significant 2.4- and 2.6-fold increase in PNAs in Aab^+^ children and T1D-onset patients, respectively, compared with healthy controls (Figure 4A). Human blood PNAs, representing aggregates between a single neutrophil and one or more platelets, were confirmed by ImageStream analysis (Figure 4B). The PNA levels were dramatically lower in children within 1 year of T1D diagnosis, representing 14% (*P* < 0.0001) and 36% of the levels at T1D onset and in normal healthy controls, respectively. A negative correlation was found between the percentage of PNAs and the number of circulating neutrophils (*P* = 0.0041; *r* = –0.3035; Figure 4C); no correlation was observed for corresponding platelet levels (*r* = –0.1012; Figure 4D). Thus, donors characterized by a lower circulating neutrophil count (high risk of developing T1D; ref. 12) were also those in whom a higher proportion of the circulating neutrophils were aggregated with platelets. Overall, these data indicate that mean circulating PNAs are significantly increased during T1D development in humans.

### Human PNAs contain 2 subpopulations of CD41^+^ platelets expressing CD62P.

We identified 2 subpopulations of CD41^+^ platelets within human blood PNAs: CD41 intermediate (CD41^int^) and CD41^hi^ (Figure 4E). Compared with healthy controls, the CD41^hi^ subpopulation in PNAs of Aab^+^ children exhibited a 3.6-fold higher expression of CD62P (*P* < 0.05; Figure 4, F–H); in parallel, CD62P levels appeared to be reduced on CD41^int^ PNAs (~12% of controls) (Figure 4I). T1D-onset donors showed an opposite trend, with a 1.6-fold increase in CD41^int^ PNAs (Figure 4I). These findings suggest that a subpopulation of platelets in the PNAs of Aab^+^ donors are hyperactivated.

## Discussion

In this study, we discovered that PNAs in the peripheral blood of NOD mice increase during T1D development. Mean PNA levels spiked at 4 weeks and 10–12 weeks of age, as well as at T1D-onset, and correlated with histological evidence of increased inflammation (4 weeks) and a higher frequency of host pancreatic islets demonstrating leukocyte invasion — i.e., destructive insulitis at 10–12 weeks and at T1D onset. Interestingly, individual mice at most time points, as well as at T1D-onset, segregated into 2 subgroups identified by high or low PNA levels. Similarly, we found a significant increase in the mean circulating levels of PNAs in Aab^+^ pre-T1D children and in new-onset T1D pediatric patients, with subpopulations in each cohort characterized by high to low PNA levels. In contrast, there was a dramatic decline in blood PNA levels already by < 1 year after T1D onset, consistent with low PNA levels previously reported for children at 0.5–4.3 years after T1D onset (42). These lower PNA levels could be due to NET release (NETosis) induced by hyperglycemia (49), resulting in the rupture of the cell membrane of neutrophils and, hence, the inability to identify cell-surface antigens. Alternatively, platelet microparticles released from activated platelets, as previously reported for autoimmune primary Sjögren’s syndrome (50), could reduce CD41 expression and, thus, PNA detection. Overall, our findings strongly indicate that elevated PNA levels in peripheral blood potentially represent a novel innate immune biomarker of T1D autoimmunity.

Compared with resting platelets, the platelets in circulating NOD PNAs were highly activated, as indicated by strong staining for the platelet activation marker CD62P. Mechanistically, CD62P expressed on the surface of activated platelets binds to PSGL1, the cognate receptor on the surface of neutrophils (22, 24). This platelet-neutrophil interaction activates neutrophils and can signal intracellular pathways essential for NETosis (22, 51). Alternatively, activated platelets help neutrophils to adhere to vascular endothelium and secrete chemokines required for neutrophil extravasation and migration into tissues, as previously demonstrated in other models of inflammation (22, 27, 52, 53). Our data suggest that PNAs represent a niche that promotes platelet-induced activation of neutrophils and neutrophil migration into the pancreas via the conventional platelet-derived chemokines PPBP/CXCL7 and CXCL4 (platelet factor 4 [PF4]) (52). Thereafter, at least some activated neutrophils release NETs, resulting in the retention of NET products in the islet/insulitis microenvironment, as demonstrated by immunofluorescence staining of pancreas sections (Figure 2). This interpretation is supported by the profile of temporally increased blood PNA levels (Figure 1D), which correlate with increased islet/insulitis PNAs (Figure 1G) and an increased frequency of islet-associated NET products (MPO and CitH3) (Figure 2, B and C). Our demonstration of NET- and histone-induced damage to isolated normal islets in vitro (Figure 3, A, C, and E) further suggests that NET products are likely to contribute to β cell demise during T1D pathogenesis as nonspecific mediators of tissue damage, supplementing the major destructive mechanisms of autoantigen-specific T cells. It should be noted that, irrespective of NOD mouse age or T1D-onset, we found only occasional islet-associated, NET-releasing neutrophils. We reason that NETs are difficult to localize in pancreas tissue because NET DNA is likely to be rapidly degraded by DNases released locally by nearby exocrine tissue (54). Overall, increased circulating PNAs appear to correlate with the initiation of T1D autoimmunity in young NOD mice (15), with a critical pivot in disease progression and local recruitment of activated neutrophils at both 10–12 weeks and the clinical onset of T1D.

The islet-associated NET products observed in NOD pancreas suggested a possible pathogenic role in T1D, as previously implicated in a mouse model of lung injury (55). Our in vitro studies revealed that cell-free calf thymus histones and NETs released by activated neutrophils were toxic for islets and that they induced extensive or focal damage at the islet periphery, respectively. Histone tails carry a high positive charge, and their toxicity for human and mouse islets could be due to electrostatic interactions resulting in the formation of pores in the plasma membrane of β cells and subsequent apoptosis (56, 57). While the specificity of histone- and NET-induced injury for β cells was not addressed in our in vitro experiments, our immunofluorescence studies of NOD pancreata identified NET-derived citrullinated histones within islets, suggesting their likely interaction with β cells during T1D disease progression. Moreover, we attribute the protective effect of mCBS to its anionic properties and the neutralization of the high positive charge carried by histones (47). Thus, mCBS most likely protected mouse and human islets in vitro by preventing the interaction between calf thymus histones or NET-associated/derived histones and islet cell surface membranes, as similarly reported for other in vitro systems and acute NET-mediated pathologies in vivo (47). While mCBS activity in vitro demonstrated proof of principle that histone/NET-mediated islet damage can be successfully inhibited, we observed that in vivo treatment of NOD mice with mCBS failed to significantly ameliorate the incidence of T1D. Similarly, treatment of NOD mice with inhibitors of MPO and NE (NET products) has not prevented T1D development (58). Together, these findings support the understanding that neutrophils alone do not induce T1D diabetes in NOD mice; instead, neutrophils (via platelet activation and NET release) provide a secondary or accessory destructive process that can contribute to β cell death during T1D pathogenesis — i.e., supplementing β cell loss due to T cell–mediated mechanisms. Recently. Sodre et al. (59) reported that treatment of NOD mice with a pan peptidyl arginine deiminase (PAD) inhibitor prevented T1D. However, this therapeutic approach inhibits PAD4, which plays a critical role in NETosis and also other PADs, including PAD2, which modulates the differentiation of Th2 T cells. Such studies have, therefore, highlighted an important role for protein citrullination, rather than NETs alone, in T1D development (59, 60). Our findings implicate the potential for direct effects of NET-derived histones on islet damage in T1D, in contrast to the indirect proinflammatory effects of NE (61) and possible indirect inflammation associated with rapid clearance of NET DNA (62). Neutrophil activation by platelets within PNAs could, therefore, promote the release of NETs and NET-derived proteins (including citH3, NE) that contribute to T1D autoimmunity and β cell destruction. Collectively, our findings indicate that elevated circulating PNAs could potentially represent both a pathogenic and innate immune biomarker of T1D development.

Like NOD PNAs, we discovered that PNAs in pre-T1D and T1D-onset children contained activated platelets, with a subset of PNAs in Aab^+^ children characterized by “hyperactive” platelets expressing high levels of CD62P. Significantly, a relationship between platelet hyperreactivity and T1D development before the clinical onset of hyperglycemia has not previously been reported. Instead, platelet activation has generally been investigated in long-term T1D, specifically in the context of secondary micro- and macrovascular complications, including nephropathy and cardiovascular disease (34, 35, 42, 63–66). Extrapolating from our cross-sectional studies of PNAs in NOD mice, we suggest here that elevated circulating PNAs in Aab^+^ children and children at T1D onset should be further evaluated as a candidate innate immune biomarker of T1D disease progression. Based on our correlative analyses, heightened PNA levels provide a rational explanation for the mechanism by which the number of neutrophils decline in the circulation in pre-T1D and T1D-onset children (12, 14) and for why soluble P-selectin (CD62P) is increased in the circulation of patients with newly diagnosed T1D (41). It is also possible that elevated PNA levels may help to explain why neutrophils from T1D individuals are more susceptible to NETosis (49) and why circulating NET DNA and neutrophil enzymes are increased in recent-onset T1D (13). We suggest that PNAs may either lead to NETosis in peripheral blood and/or to neutrophil mobilization to the pancreas and local islet-associated NETosis. Moreover, we speculate that the downstream properties of neutrophils that undergo activation in PNAs could be determined by the differences we observed in the status of platelet activation, potentially depicting distinct platelet subtypes (67). Future studies addressing causality could systematically block different ligand-receptor interactions between platelets and neutrophils and potentially identify a T1D-relevant pathway of neutrophil activation. Targeting P-selectin therapeutically appears to be feasible, as a number of emerging anti–P-selectin agents are being evaluated in clinical trials for treatment of a range of vascular disorders (68). Of interest, our data suggest that activated platelets may indirectly contribute to T1D autoimmunity. We therefore suggest that combinations of T cell intervention strategies with drugs that inhibit platelet hyperresponsiveness and/or that neutralize histone toxicity warrant testing in at-risk children with high blood PNA levels, to assess whether asymptomatic T1D disease progression can be impeded.

Recently, there has been growing support for heterogeneity in human T1D disease and the concept of different endotypes, based on different intraislet leukocyte profiles revealed by IHC staining of T1D human pancreata and different gene sets identified by RNA-Seq of whole blood from recent-onset T1D donors (69–73). Such endotypes are characterized by different cellular immunotypes, currently defined by B cells and neutrophils, and accompany a principal T cell–mediated autoimmune response (70, 71). We speculate that PNAs could possibly act as a biomarker for a neutrophil-associated T1D endotype and as a potential aid for selecting combinations of therapeutics for personalized T1D prevention or treatment. Interestingly, NOD mice have been found to exhibit either acute or chronic changes in blood glucose levels before T1D onset, suggesting that T1D development in the NOD mouse model may also be heterogeneous (74), a concept supported by our PNA studies. Ultimately, the potential for PNAs to be predictive biomarkers of T1D will require further clinical evaluation. In particular, longitudinal studies in at-risk children could potentially determine whether the combination of blood PNA levels, the number/titres of T1D-specific autoantibodies, and glucose tolerance testing (73, 75) more sensitively predicts T1D in children and identifies individuals with an underlying neutrophil-associated endotype.

## Methods

### Mice and T1D monitoring.

Female NOD/Lt mice (4–5 weeks old) and pregnant NOD/Lt (E14) females were obtained from the Animal Resource Centre (ARC; Perth, Australia). B6.SJL-Ptprc^a^Pepc^b^/BoyJ (B6SJL) mice (nonautoimmune controls) and BALB/c mice (for in vitro studies of histone and NET treatment) were sourced either from the Australian Phenomics Facility, The John Curtin School of Medical Research, ANU (Canberra, Australia), or from ARC (Perth, Australia). The mice were housed under specific pathogen–free conditions at the Australian Phenomics Facility, ANU, in autoclaved individually ventilated cages (up to 5 mice/cage), containing aspen bedding and nesting material for enrichment. All mice were maintained with irradiated standard rodent chow and water ad libitum with environmental conditions set at ~19°C and an automated 12-hour light/dark cycle. Clinical onset of diabetes was determined by measuring urine glucose with Multistix reagent strips (Bayer) and confirmed by measuring nonfasting blood glucose levels in tail vein blood using a MediSense glucometer (Abbott Laboratories). Hyperglycemia (T1D-onset) in this study was defined as 2 consecutive blood glucose readings ≥ 14 mmol/L.

### Human volunteers for blood samples.

Children (5–16 years of age) with an increased risk for T1D were identified by having first- or second-degree relatives with T1D; these at-risk children were Aab^–^ (*n* = 19) or Aab^+^ (*n* = 14) participants in the T1D TrialNet Pathway to Prevention Study TN01 in Milan (Italy) or in Canberra (Australia) (Supplemental Table 1) (76). Children (5–16 years of age) at T1D onset (~ 10 days after diagnosis [after initial insulin injection]; *n* = 34) or with recent-onset T1D (duration < 1 year; *n* = 6) were recruited prior to discharge from the Pediatrics Department, Ospedale San Raffaele, or from the Pediatric and Adolescent Diabetes Clinic, The Canberra Hospital (Canberra, Australia) (Supplemental Table 1). There was no overlap between pediatric cohorts. Healthy pediatric volunteer controls (5–16 years of age; *n* = 46) were recruited with consent from local schools (Canberra, Australia) and from a group of healthy children hospitalized for elective surgery (pre-anesthetic; Orthopedic Pediatric Department, San Raffaele Hospital, Milan, Italy) (Supplemental Table 1). Healthy adult volunteers were used for assay controls. The exclusion criteria included: chronic inflammation, infection (including sepsis), non-T1D autoimmune disease, recent surgery/inflammation, use of nonsteroidal anti-inflammatory drugs/aspirin (within 1 week of blood sample), treatment with systemic glucocorticoids, and non-T1D endocrine disorders (e.g., pituitary hormone deficiency). Race and ethnicity information for volunteers was not collected, as it was not included in the human ethics approvals.

### Islet isolation and preparation of insulitis leukocytes.

Mouse pancreatic islets were isolated by intraductal infusion of collagenase P (2.5 mg/mL; Roche Diagnostics) in isolation medium (20 mM HEPES-buffered HBSS/0.15% BSA (30063-572, Invitrogen)/1% DNase (D5025, MilliporeSigma)/antibiotics (penicillin G [0.06 mg/mL; 156065, MP Biomedicals]/streptomycin (0.10 mg/mL; S6501, MilliporeSigma)/neomycin (0.10 mg/mL; N-6386, MilliporeSigma) supplemented with prostaglandin I_2_ (200 nM; P6188, MilliporeSigma). The islets were handpicked with the aid of a dissecting microscope (Kyowa Optical SDZ-P) (77–80). For the isolation of insulitis leukocytes, islets harvested from individual donors (NOD; *n* = 5–15 NOD donors/group) or pooled donors (B6SJL; *n* = 12 donors/4 experiments/group) were mashed over a sieve (70 μm; 352350, Falcon, Corning Inc.) in 100 μL 5% fetal calf serum (FCS; SH30071.03, Hyclone Labs)/Ca^2+^Mg^2+^-free phosphate-buffered saline (PBS; D8537, MilliporeSigma). The insulitis leukocytes were harvested from the medium, and noninsulitis islet cells were retained by the sieve. Human islets of Langerhans allocated for research use were provided by St. Vincent’s Institute of Medical Research, Melbourne, Australia.

### Flow cytometry.

Mouse blood samples were collected from the retroorbital sinus into buffered saline (115 mM NaCl)/13.6 mM tri-sodium citrate/11.1 mM glucose containing 0.9 mM EDTA (BSCG buffered anti-coagulant) (81), 100 μL blood/200 μL anticoagulant (≥ 6 weeks of age for NOD and B6SJL mice), and 50 μL blood/100 μL anticoagulant (for young NOD mice at 2–4 weeks of age), *n* = 10–26 NOD mice/group and *n* = 52 B6SJL/group. Human blood samples (4–8 mL) were collected into ACD-B vacutainers (13.2 g/L trisodium citrate, 4.8 g/L citric acid, 14.7 g/L dextrose citrate; Greiner Bio-One; catalog 455094 or 456094) using a 23 G or 21 G needle. In total, 1 mL of blood was diluted 1:4 with Ca^2+^Mg^2+^-free PBS (hereafter PBS). To detect mouse PNAs, whole blood (50–100 μL) was diluted 1:1 with PBS and was maintained at room temperature without centrifugation to prevent inadvertent platelet activation during the staining procedure. The blood cells were transferred to a 96-well plate (75 μL/well; Cellstar, Greiner Bio-One, 650 180), incubated with rat anti–mouse CD16/CD32 (Fc block; Supplemental Table 2) and then stained at room temperature with fluorescently labeled anti-mouse CD45.1 (common leukocyte antigen; APC), CD11c (DCs; PECF594), CD11b (myeloid cells; AF700), Ly6C (inflammatory macrophages/neutrophils; expressed at lower levels on NOD cell populations than for conventional mouse strains; BV605; refs. 82, 83), Ly6G (neutrophils; PE-Cy7), and CD41 (platelets; FITC), each diluted at 1:100 (in 150 μL; Supplemental Table 2). Each sample (150 μL) was transferred to FACS mini tubes (Axygen), fixed (1:1.3) with Fixation and Permeabilization solution (BD Cytofix/Cytoperm, 51-2090KZ, BD Biosciences) and then analyzed on a LSRII flow cytometer (Becton Dickinson). FlowJo software (v10.0.7, Tree Star Inc.) was used to determine the percentage of neutrophils (Ly6G^+^) aggregated with platelets (Ly6G^+^CD41^+^ [PNAs]; see Supplemental Figure 1). The percentage of PNAs in adult B6SJL mice served as nonautoimmune controls in each assay. To detect human PNAs, diluted (1:4) whole blood (50–100 μL/well of a U-shaped 96-well plate [Cellstar, 650180, Greiner Bio-One] or 100 μL/microcentrifuge tube [Sarstedt AG & Co. KG, 72.706]) was stained in the dark for 15 minutes with anti–CD15-V450, anti–CD16-PE-Cy, and anti–CD41-PE (Supplemental Table 3). The human samples were then fixed with 8% paraformaldehyde (PFA) (P6148; Sigma-Aldrich, Merck; 50–100 μL/well) for 15–20 minutes and transferred to FACS minitubes. Finally, human samples were diluted 1:9 with PBS and incubated for 1 hour in the dark to promote lysis of RBCs. In some experiments, platelet activation in mouse and human PNAs was examined by including anti-CD62P (P-selectin; see Supplemental Tables 2 and 3) in the staining mix and CD41 FMO (fluorescence minus one) control was used to set the gate for CD41^+^ cells. Samples were run on the same day as staining using a LSRII flow cytometer (Becton Dickinson) or BD FACSCanto II (BD Biosciences) flow cytometer. FlowJo software (v10.0.7) or FCS Express (v6; De Novo Software) was used to determine the percentage of neutrophils (CD15^+^CD16^+^) with bound platelets (CD15^+^CD16^+^ CD41^+^; see Supplemental Figure 8), representing the percentage of PNAs. For multispectral imaging of PNAs, diluted mouse blood was treated with Fc block, stained with anti–Ly6G-PE-Cy7 and anti–CD41-FITC, fixed (1:2 with BD Cytofix/Cytoperm) and diluted (1:9) prior to analysis using an ImageStream Cytometer (Amnis Corp). Human PNAs were similarly analyzed using ImageStream^X^ (Amnis, Merck) after staining diluted human blood with anti–CD15-V450 (dilution 1/24), anti-CD16-PE-Cy7 (dilution 1/24), and anti-CD41-PE (dilution 1/12), with fixation and dilution in PBS (as for routine flow cytometry).

### Neutrophil and platelet counts.

Human blood samples from the same donor were drawn sequentially into citrate anticoagulant (for PNA measurement) and EDTA anticoagulant (BD Biosciences, 368856 vacutainers) for complete blood counts (CBCs, including neutrophil and platelet counts). CBCs were analyzed using a Sysmex XE-2100 automated hematology analyzer at the San Raffaele Hospital. Mouse blood cell counts were determined from retroorbital blood samples (50 μL [2-week-old mice only], 200 μL) collected into EDTA-tubes (Microvette, 20.1288, Sarstedt). The blood was diluted 1/4 (for samples from 2-week-old mice) or 1/2 with 5% FCS/PBS and analyzed using the ADVIA 2120i Hematology System (Bayer Healthcare).

### Immunofluorescence staining of NETs.

Neutrophils were isolated from human peripheral blood of healthy donors using the MACSxpress Neutrophil Isolation Kit (103-104-434, Miltenyi Biotec). To identify NET-derived proteins by immunofluorescence microscopy, neutrophils with PMA (50 nM), CI (5 μM) (to initiate activation), or unstimulated neutrophils (1 × 10^6^ neutrophils/mL MCDB 131 medium [10372-019, Thermo Fisher Scientific])/0.5% FBS (F9423, MilliporeSigma) were transferred to individual glass coverslips (13 mm, 0117530, Marienfeld; 50 μL/coverslip) in a 24-well culture plate (3524, Costar, Corning Inc; 1 coverslip/well). After culture in 5% CO_2_, 95% air for 4 hours, the cells were fixed with 4% PFA (500 μL/well), and the plate was stored at 4°C (84). For immunofluorescent staining of NET proteins, the PFA was removed, and the cells were gently washed with PBS and blocked with 5% cold water fish gelatin (G7041, MilliporeSigma)/5% BSA Fraction V (Invitrogen)/0.05% TWEEN 20 (P1379, MilliporeSigma)/0.05% Triton X-100 (BDH, 30632.6P, Merck)/2% donkey serum (D9663, MilliporeSigma) in PBS for 30 minutes at room temperature to reduce autofluorescence (500 μL/well) (85). A staining mix of goat anti–human/mouse MPO (Supplemental Table 4) and rabbit anti–histone H3 (citrulline R2 + R8 + R17; Supplemental Table 4) was applied to the cells on each coverslip (50 μL/ coverslip) and incubated at 37°C in a humidified tray for 1 hour. After washing with PBS, aided by gentle rotation on a plate shaker (Labinco LD-45, Lomb Scientific Pty Ltd.), a staining mix of the secondary antibodies donkey anti–goat IgG-AF488 and donkey anti–rabbit IgG-AF568 (Supplemental Table 4) was applied (50 μL/coverslip) and incubated at 37°C for 30 minutes (84). After washing with PBS, the cells were stained with Hoechst 33342 (MilliporeSigma; 1/1000) for 10 minutes with gentle rotation and then washed. The coverslip with stained cells was mounted onto a slide (with cells on underside of coverslip) using ProLong Diamond antifade mountant (P36961, Invitrogen). Images were acquired using an inverted fluorescence microscope (Zeiss AxioObserver).

### Treatment of isolated islets with NETs.

NETs were produced in vitro by stimulating human neutrophils with PMA or CI. PMA activates protein kinase C (PKC) and stimulates NADPH oxidase–dependent NETosis; CI-NETosis is NADPH independent, requiring intracellular calcium for inducing histone citrullination by peptidylarginine deiminase (86). Isolated human neutrophils were incubated in 6-well plastic plates (140675, Thermo Fisher Scientific), 3.5 × 10^6^/1.5 mL MCDB 131 medium/0.5% FBS with PMA (50 nM) or CI (5 μM) for 4 hours in a humidified gas phase of 5% CO_2_, 95 % air at 37°C. Unstimulated neutrophils were incubated with culture medium only. The wells were then gently washed with PBS, and the NET DNA in selected wells was treated with the restriction enzyme *Alu*I (4 U/mL). The DNA content of the NET-rich supernatant was measured using 50 nM SYTOX Green (S7020, Invitrogen; 95 μL/well); the green fluorescence was measured using a multimode plate reader (TECAN Infinite M200 PRO) and a standard curve generated from calf thymus histones (0.19–100 μg/mL) (47). The DNA content of PMA-NETs, CI-NETs, and unstimulated neutrophils was 0.422–0.997 μg/mL, 0.104–0.784 μg/mL, and 0.000–0.185 μg/mL, respectively. Isolated mouse islets were added to the remaining washed wells (50 islets/well) in RPMI 1640 (R0883, MilliporeSigma)/10% FCS (Hyclone; 2 mL/well) with or without mCBS (a polyanionic drug that protects against histone-induced cell damage; 100–800 μg/mL; ref. 47) and cultured in 5% CO_2_, 95 % air at 37°C for 17 hours. In parallel, some islets were cultured with calf thymus histones (800 μg/mL; H9250, MilliporeSigma) alone, serving as a positive control for NET-associated histones. After culture, the medium was removed from the islets, and the islets were labeled with Calcein-AM (1 μM in PBS; 100 μL/well; C3100MP, Invitrogen, Molecular Probes) and Propidium iodide (PI; 10 μg/mL in PBS; 100 μL/well; 556463, BD Biosciences) for 30 minutes in 5 % CO_2_, 95 % air, to identify islet cell viability and death, respectively. After labeling, 1.8 mL RPMI 1640/10% FCS was added to each well, and the islets were transferred to glass bottom dishes (29 mm, D29-20-1.5-N, Cellvis) for imaging by confocal microscopy. To confirm NET release, neutrophils from each experiment (0.75 × 10^6^/500 μL of MCDB 131 medium/0.5% FBS) were also activated with PMA (50 nM) or CI (5 μM) for 4 hours at 37°C in glass bottom dishes (Cellvis) to optimize imaging. After washing with PBS, NET DNA was labeled with SYTOX Green (5 μM) and imaged with an inverted fluorescence microscope (Zeiss AxioObserver).

### Treatment of isolated islets with calf thymus histones.

Isolated mouse islets (50 islets/2 mL per well) were cultured in 6-well plastic culture plates (657175, Greiner) with calf thymus histones (100–800 μg/mL; H9250, MilliporeSigma) for 2–48 hours in RPM1 1640 medium/10% FCS, at 37°C in 5% CO_2_, 95% air. In additional experiments, isolated mouse islets were cultured with histones (400–800 μg/mL) and mCBS (100–800 μg/mL) for 2–18 hours. Humans islets (~700–850 islet equivalents/2 mL per well; provided by the Susan Alberti Islet Isolation Program, St. Vincent’s Medical Research Institute, Melbourne, Australia) were also cultured with histones (400–800 μg/mL), with or without mCBS (100–800 μg/mL), for 18 hours. After culture, the medium was removed and the islets were labeled with Calcein-AM (1 μM) and PI (10 μg/mL) for 30 minutes in 5% CO_2_, 95% air. After labeling, the islets were transferred to glass bottom dishes (29 mm, Cellvis) in 2 mL culture medium for imaging by confocal microscopy.

### Histology and immunofluorescence staining of pancreas sections.

Mouse pancreata were fixed in 10% neutral-buffered formalin, and paraffin sections (4 μm thick) were stained with H&E. For labeling by immunofluorescence, unstained pancreas sections (*n* = 3–5 NOD pancreata/group; *n* = 3 B6SJL pancreata/group) underwent antigen retrieval using citrate buffer (pH 6)/heat before being permeabilized with 0.5% Triton X 100 (Merck). The permeabilized sections were blocked with 5% cold water fish gelatin (MilliporeSigma)/5% BSA (Invitrogen)/0.05% TWEEN 20 (MilliporeSigma)/0.05% Triton X-100 (Merck)/2% donkey serum (MilliporeSigma) in PBS (85) to reduce red cell autofluorescence and background staining. The sections were then incubated with unlabeled anti-mouse MPO, anti–histone H3 or anti-Ly6G (Supplemental Table 4) overnight at 4°C. After washing with PBS, the sections were incubated with donkey anti–goat IgG-AF568 or donkey anti–goat IgG-AF488 (for MPO), donkey anti–rabbit IgG-AF568 (for histone H3), or donkey anti–rat IgG-AF488 (for Ly6G) (Supplemental Table 4), at room temperature. Background staining was determined using sections stained only with the secondary antibody. The sections were washed in PBS and Hoechst 33342 (H1399, MilliporeSigma; 1/10,000 or 1 μg/mL) was applied to label DNA. After washing, Trueblack (Biotium; 1/20 in 70% ethanol) was applied for 30 seconds to reduce autofluorescence (87). The slides were washed, and each slide was mounted using a 1.5H cover glass (Marienfeld, 0107222) and antifade mounting medium (ProLong Diamond). Immunofluorescence staining was observed using a Zeiss AxioObserver inverted microscope, and images were collected using the Apotome2 attachment (Zeiss), which removes unfocused light; the apotome images were merged using ZEN software (Zeiss). Quantitative analysis of MPO, CitH3, and Ly6G positive islets was done by counting the number of total islets and expressing the number of MPO^+^, CitH3^+^, or Ly6G^+^ islets in each section as a percentage of total islets.

### Confocal microscopy.

NET- or calf thymus histone–treated islets were imaged at 30 μm intervals (*Z* stack; *n* = 3–7 focal planes/islet) using a Leica SP5 confocal microscope. The percentage of PI^+^ islet area was measured for each focal plane using ImageJ software (NIH; v2.0.0). The first focal plane of islets cultured with NETs contained PI^+^ neutrophils and was, therefore, excluded from quantitative analyses.

### Statistics.

Comparisons between data sets were performed using GraphPad Prism software. For mouse studies, the nonparametric Kruskal-Wallis test with Dunn’s multiple comparisons test was used to compare blood PNAs, islet PNAs, and CD45^+^ leukocytes between mouse donors of different age/T1D stage or strains and also for analysis of islet cell death after histone or NET treatment. One-way ANOVA with Tukey’s multiple comparisons test was used to compare CD62P levels on platelets/neutrophils/PNAs and the percentage of islets positively stained for NET products or neutrophils. Fisher’s exact test was used to compare islet histological scores between pancreas groups from NOD donors at different ages/T1D stage or between an NOD pancreas group and the B6SJL control group. For human data, the nonparametric Kruskal Wallis test with Dunn’s multiple comparisons test was used to compare the percentage of PNAs and CD62P MFI (median fluorescence intensity) between pediatric groups. Wilcoxon test was used to compare the CD62P MFI between different subsets of PNAs (CD41^hi^ and CD41^int^) within each group. The nonparametric Spearman correlation test was used to evaluate the The nonparametric Spearman correlation test was used to evaluate the relationships with total neutrophil and platelet counts. A *P* value less than 0.05 was considered statistically significant. Graphics were prepared using GraphPad Prism v8.4.3.

### Study approval.

This study protocol and consent documents were approved by appropriate independent ethics committees (IRBs), including ACT Health Human Research Ethics Committee (ACT ETH.3.17.052; REGIS 2019/ETH11059) and the Human Research Ethics Committee of ANU (no. 207/336). Relatives of patients with T1D and Aab^+^ children were enrolled in the TrialNet Pathway to Prevention Trial (TN01) at Ospedale San Raffaele, Milan, Italy (IRB NHPROT32803-TN01) and The Canberra Hospital, Canberra, Australia (ETH.8/07.761). All participants or parents of children provided written consent or signed assent forms. For isolated human islets, informed consent from next of kin was obtained. Mouse studies were approved by ANU Animal Experimentation Ethics Committee (A2017/07).

## Author contributions

SKP, FV, DJB, RF, YS, YT, RW, MAS, JG, and AG performed the experiments and analyzed data. EB, MB, AP, ARAL, KJB, LAC, EEG, HET, and BHC provided human samples and/or contributed to the study design. CJS, CRP, AP, and MB analyzed data and/or conceived the study. CJS, SKP, FV, EB, and AP prepared the manuscript. SKP, EEG, FV, RF, YS, YT, RW, ARAL, JG, MAS, CRP, HET, and BHC reviewed the manuscript.

## Supplementary Material

Supplemental data

## Version 1 01/25/2022

Electronic publication
