# Supplementary material for: Circulating platelet-neutrophil aggregates characterize the development of type 1 diabetes in humans and NOD mice
Source: JCI Insight. 2022 Jan 25;7(2):e153993. doi: 10.1172/jci.insight.153993 (PMC8855805; doi:10.1172/jci.insight.153993)
Supplement: Supplemental data [file jciinsight-7-153993-s079.pdf]

SUPPLEMENTAL MATERIAL

Supplemental Method

Treatment of NOD mice with mCBS. NOD female mice at 2 weeks or 10-12 weeks of age were treated with mCBS (10 mg/ml stock in saline) at 50 mg/kg/day i.p. or 5 µl saline (diluent control)/g body weight/day i.p. for 6 weeks. The mice were weighed 1 x (adults) – 2 x (neonates/young mice)/week and the volumes of mCBS and saline for injection were adjusted accordingly. Onset of T1D was monitored until the mice reached 28-29 weeks of age (see Methods section). Diabetic mice and mice that did not become diabetic by 28-29 weeks of age were ethically terminated by cervical dislocation.

Supplemental Tables

Supplemental Table 1 Demographics for pediatric donors

	HC	Aab neg	Aab pos	T1D Onset	T1D <1 yr
Age, median (n)	13.5 (46)	13.0 (19)	11.4 (17)***	11.3 (34)	12.5 (6)
Sex Female % (n)	50.0 (23)	57.9 (11)	35.3 (6)**	23.5 (8)	33.3 (2)
N Aab					
0, % (n)	100 (20)	100 (19)	0 (0)	2.9 (1)	na
1, % (n)			35.3 (6)*	17.6 (6)	na
2, % (n)			29.4 (5)**	23.5 (8)	na
3, % (n)			23.5 (4)	41.2 (14)	na
4, % (n)			11.8 (2)	14.7 (5)	na
5, % (n)			0 (0)	0 (0)	na
Type of Aab					
GAD65, % (n)			94.1% (16)***	79.4 (27)	na
ZnT8, % (n)			41.2% (7)*	73.6 (25)	na
IA2, % (n)			17.6% (3)	67.6 (23)	na
ICA, % (n)			29.4% (5)*	na	na
IAA, % (n)			29.4% (5)	26.5 (9)	na
HbA1c mmol/mol median (n)	na		32.0 (10)*	99.5 (34)	51.0 (4)
HbA1c % median (n)	na		5.1 (14)**	11.1 (34)	6.8 (4)
c-peptide ng/ml median (n)	na		1.48 (11)**	0.43 (33)	na
HLA (DR3 or DR4) % (n)	na		73.7 (14)	83.3 (10)	na

* Includes 1 repeated measure
** Includes 2 repeated measures
*** Includes 3 repeated measures

Supplemental Table 2 Antibodies used for flow cytometry analysis of mouse blood

Antibody			Source	Final conc. (dilution)
Name	Code	Stock conc.		
Rat anti-mouse CD16/CD32 (mouse Fc block)	553142	0.5 mg/ml	BD Biosciences	2.3 µg/ml (1/215)
Rat anti- mouse CD45.1-APC, clone A20	17-0453-81	0.2 mg/ml	eBioscience	2 µg/ml (1/100)
Hamster anti-mouse CD11c-PE CF594, clone HL3	562454	0.2 mg/ml	BD Horizon	2 µg/ml (1/100)
Rat anti-mouse CD11b-AF700, clone M1/70	557960	0.2 mg/ml	BD Biosciences	2 µg/ml (1/100)
Rat anti-mouse LY6C-BV605 clone AL-21	563011	0.2 mg/ml	BD Biosciences	2 µg/ml (1/100)
Rat anti-mouse Ly6G-PE-Cy7 clone IA8	560601	0.2 mg/ml	BD Biosciences	2 µg/ml (1/100)
Rat anti-mouse CD41-FITC clone MWReg30	553848	0.5 mg/ml	BD Biosciences	5 µg/ml (1/100)
Rat anti-mouse CD62P-PE clone Wug.E9	M130-2	N/A	Emfret Analytics	(1/100)

Supplemental Table 3 Antibodies used for flow cytometry analysis of human blood

Antibody			Source	Final conc. (dilution)
Name	Code	Stock conc.		
Mouse anti-human CD15-V450	561584	0.025 mg/ml	BD Biosciences	0.6 µg/ml (1/40)
Mouse anti-human CD16-PE-Cy7	557744	0.4 mg/ml	BD Biosciences	10 µg/ml (1/40)
Mouse anti-human CD41-PE	ab134372	0.02 mg/ml	Abcam	1.7 µg/ml (1/12)
Mouse anti-human CD62P (P-, selectin)				
- PerCP-Cy5.5 or	304923	0.1 mg/ml	Biolegend	2.5 µg/ml (1/40)
- AF647	304918	0.1 mg/ml	Biolegend	2.5 µg/ml (1/40)

Supplemental Table 4 Antibodies used for immunofluorescence staining

Primary and secondary antibodies			Source	Final conc.
**Name**	**Code**	**Stock conc.**		
Goat anti-mouse MPO	AF3667	0.2 mg/ml	RnD Systems	10 µg/ml (1/20)
Rabbit anti- mouse histone H3 (citrulline R2+R8+R17)	ab5103	1 mg/ml	Abcam	5 µg/ml (1/200)
Rat anti-mouse Ly6G clone IA8	551459	0.5 mg/ml	BD Biosciences	10 µg/ml (1/50)
Donkey anti-goat IgG AF568	A11057	2 mg/ml	ThermoFisher	4 µg/ml (1/500)
Donkey anti-goat IgG AF488	A11055	2 mg/ml	ThermoFisher	10 µg/ml (1/200)
Donkey anti-rabbit IgG AF568	A10042	2 mg/ml	ThermoFisher	4 µg/ml (1/500)
Donkey anti-rat IgG AF488	A21208	2 mg/ml	ThermoFisher	4 µg/ml (1/500)

Supplemental Figures

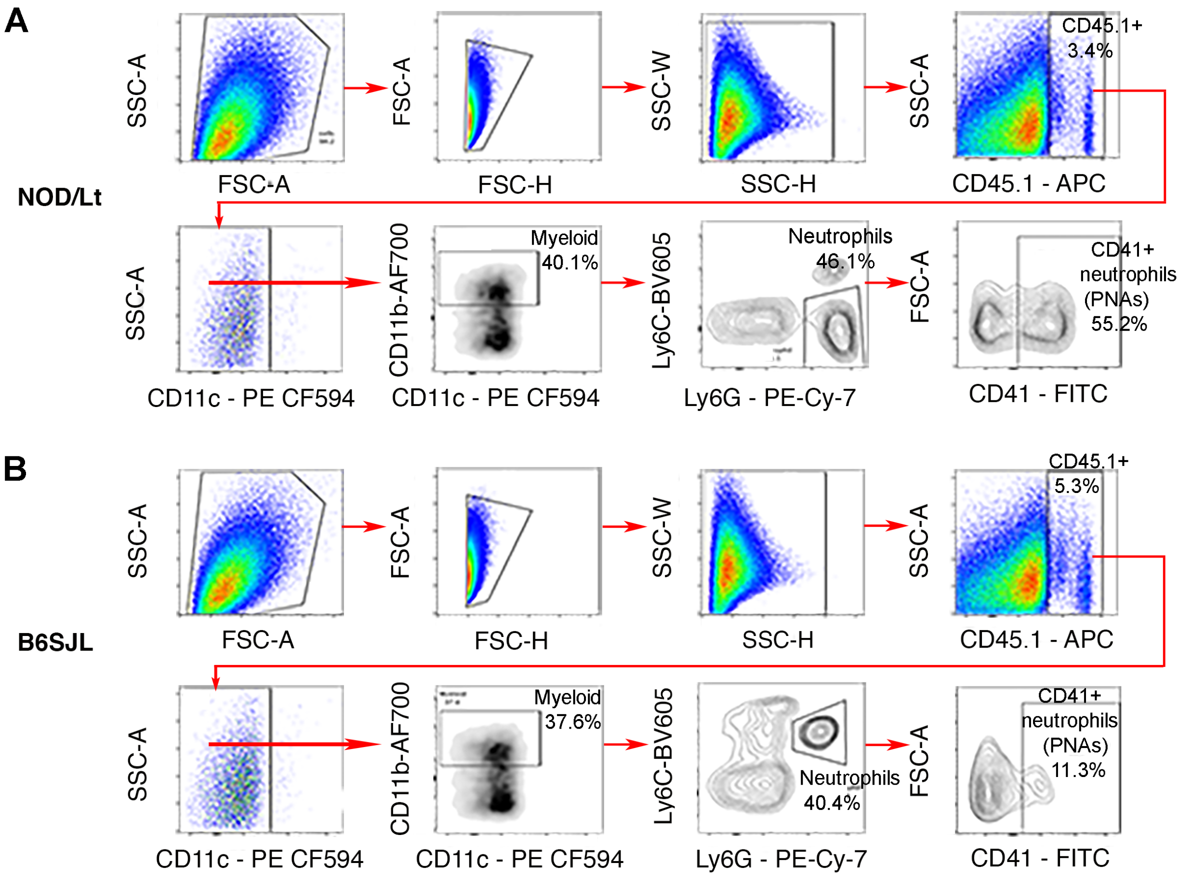

Supplemental Figure 1. Gating strategy for flow cytometry analysis of mouse PNAs. Representative FACs plots demonstrate initial identification of CD45.1+ leukocytes, followed by CD11c-/CD11b+ myeloid cells, Ly6C+/Ly6G+ neutrophils and finally, CD41 (platelet-specific marker)+ neutrophils, i.e. PNAs in (**A**) 10-12 wk pre-T1D NOD blood and (**B**) 6-8 week B6SJL blood analysed in the same assay. Note that Ly6C is expressed at lower levels on (A) NOD/Lt neutrophils (82, 83) compared to (B) B6SJL neutrophils.

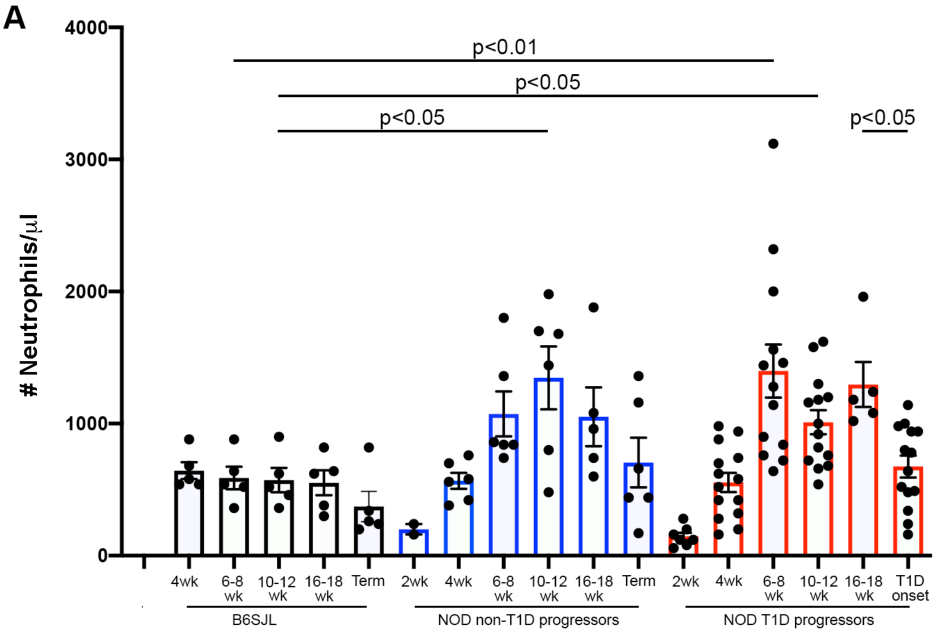

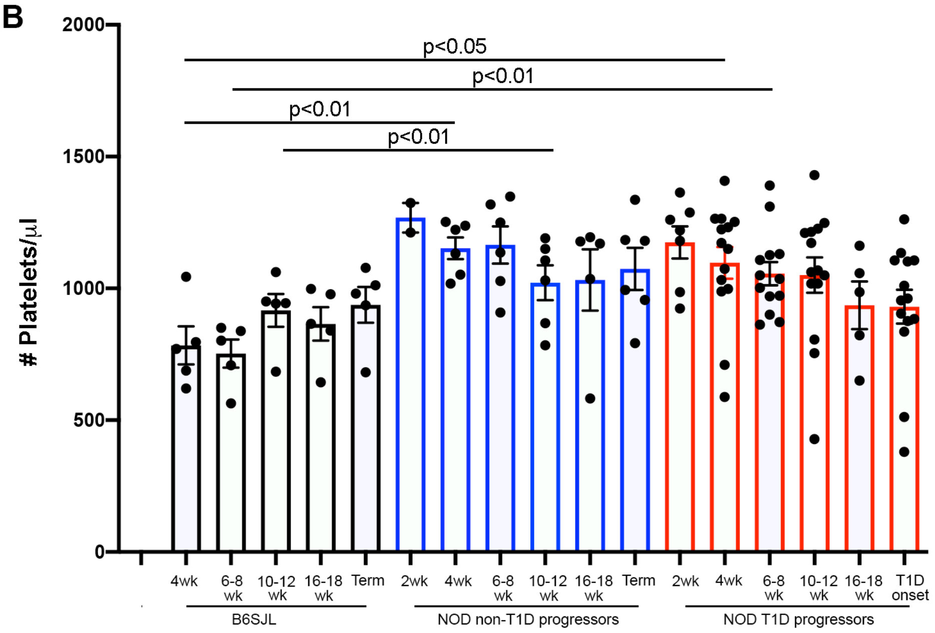

Supplemental Figure 2. Longitudinal analysis of circulating neutrophils and platelets in NOD T1D progressors and non-progressors. Bar graphs show repeated (**A**) neutrophil counts and (**B**) platelet counts in the same mice from 2wks of age (n= 9 NOD females) and from 4 wks of age (n=20 NOD females) to T1D onset or termination (25-28 weeks of age); B6SJL females were monitored from 4 wks of age only (n=5). Data shows mean ± SEM; Mixed effects analysis with Tukey's (between groups) and Dunnett's (within group) multiple comparisons tests.

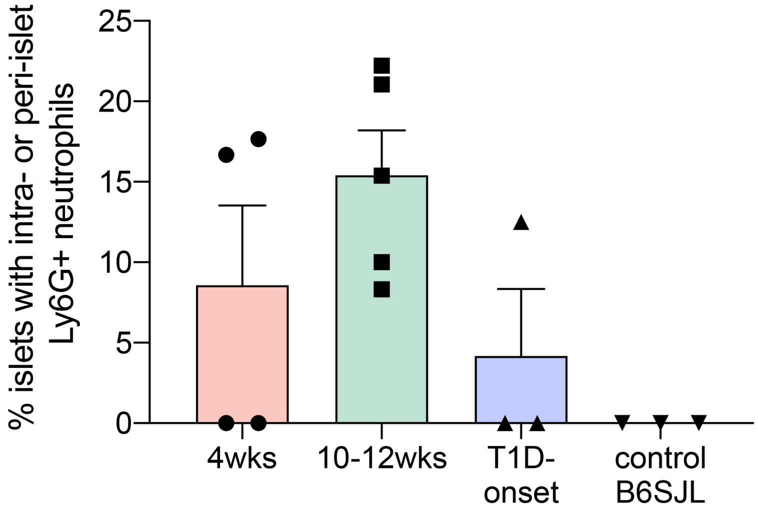

Supplemental Figure 3. Frequency of intra-islet and/or peri-islet Ly6G+ neutrophils in NOD pancreas during T1D development. Bar graphs include islets with Ly6G+ neutrophils within ~ 50 μm from the islet boundary. 1-2 pancreas sections were stained per sample. B6SJL islets were examined as non-autoimmune controls. Data shows mean ± SEM; n=3-5 pancreata/group; n=19-63 islets examined/group. No significant differences were found between the groups; One-way ANOVA with Tukey's multiple comparisons test.

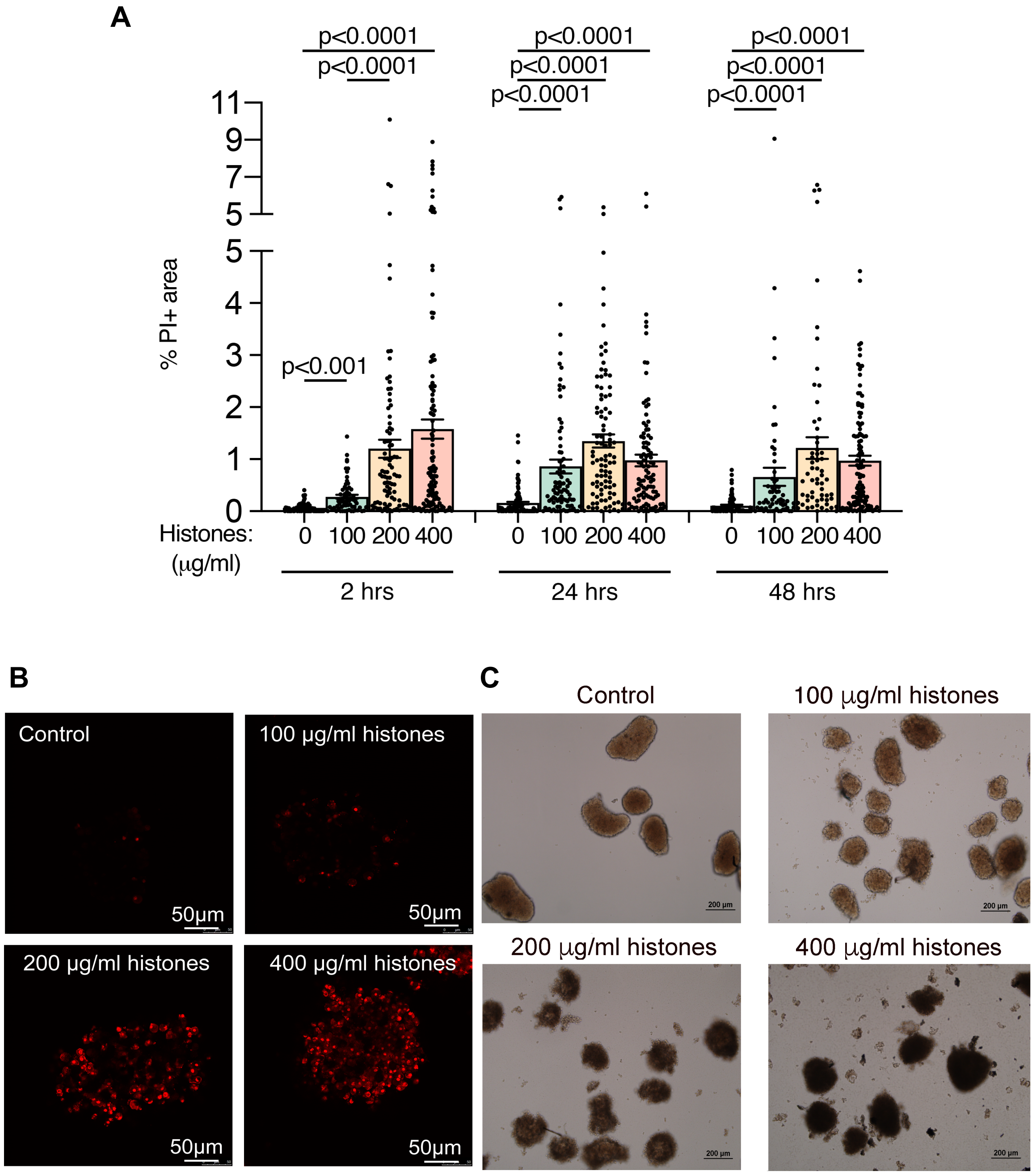

Supplemental Figure 4. Histones damage mouse islets in vitro. (**A**) BALB/c islets were cultured with calf thymus histones (100-400 µg/ml) or culture medium (control) for 2, 24 and 48 hrs (n=3 expts/group, n=15-29 islets/group/expt), labelled with PI and imaged by confocal microscopy; islet cell death (% PI+ islet area) was analysed using morphometry and Image J software. The data show mean ± SEM; non-parametric ANOVA with Kruskal-Wallis test and Dunn's multiple comparisons test. The data reveal early toxicity particularly after 2 hours of treatment with 200-400 µg/ml of histones. Representative (**B**) confocal and (**C**) macroscopic images show that BALB/c islets cultured for 2 hrs with histones showed greatly increased PI+ islet cell death (red fluorescent staining; (B)) and damaged periphery, dark appearance and surrounding cell debris after treatment with 200-400 µg/ml histones (C), compared to untreated islets. Images in (B) show the second focal planes of Z-stack (30 µm intervals). Scale bar: 50 µm in (B); scale bar: 200 µm in (C).

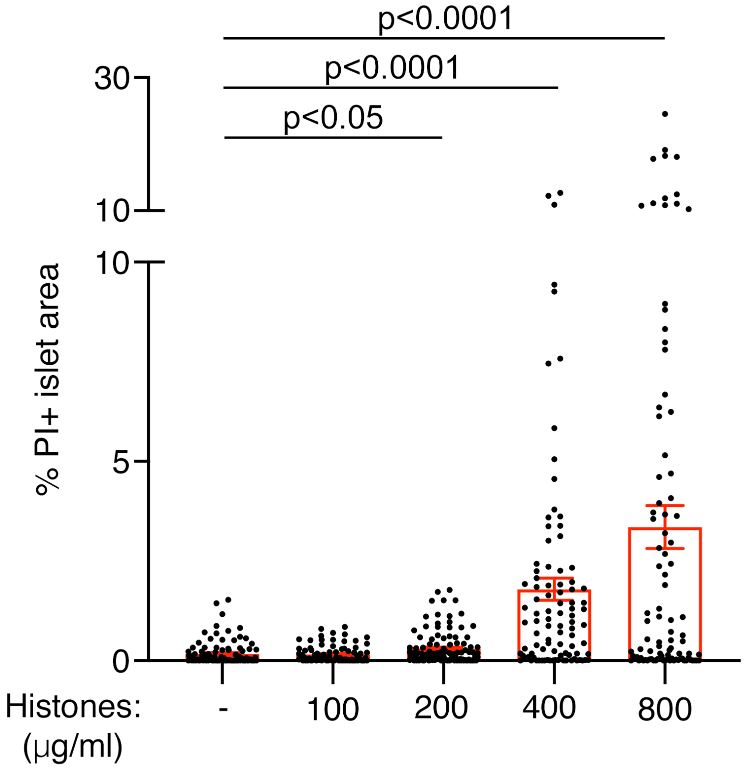

Supplemental Figure 5. Histone titration on mouse islets to identify maximal islet damage. Mouse islets treated for 2 hrs with 100-800 µg/ml of calf thymus histones show maximum islet damage (% PI+ islet area) with 800 µg/ml of histones. Data show mean ± SEM of the islets examined in each group; n=2 expts, n=18-20 islets/group, n=4-7 focal planes at 30 µm intervals examined from a Z-stack for each islet, acquired by confocal microscopy; non-parametric ANOVA with Kruskal-Wallis test and Dunn's multiple comparisons test.

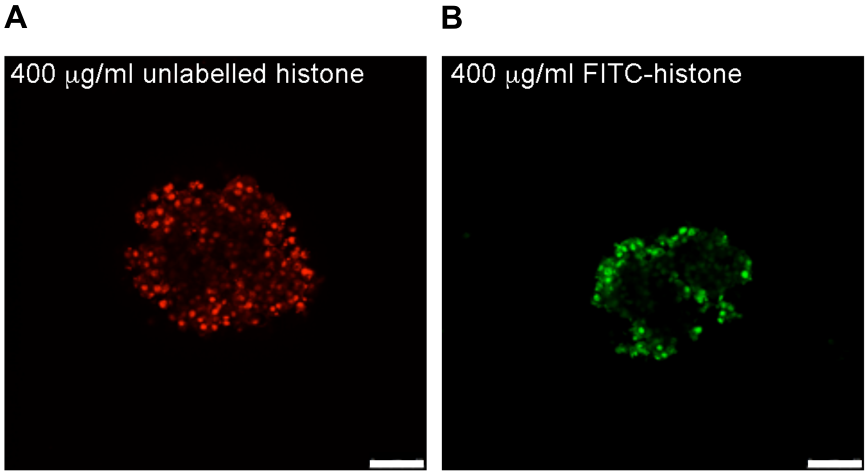

Supplemental Figure 6. Histone penetration into human islets. Representative confocal images of isolated human islets treated with 400 μg/ml unlabelled (**A**) and FITC-labelled (green fluorescent) (**B**) histones demonstrate that the peripheral staining of islets for FITC-histones (B) is consistent with the PI+ (red fluorescent) islet area routinely observed at the islet periphery after histone treatment (also see Figure 3B). Scale bar: 50 μm.

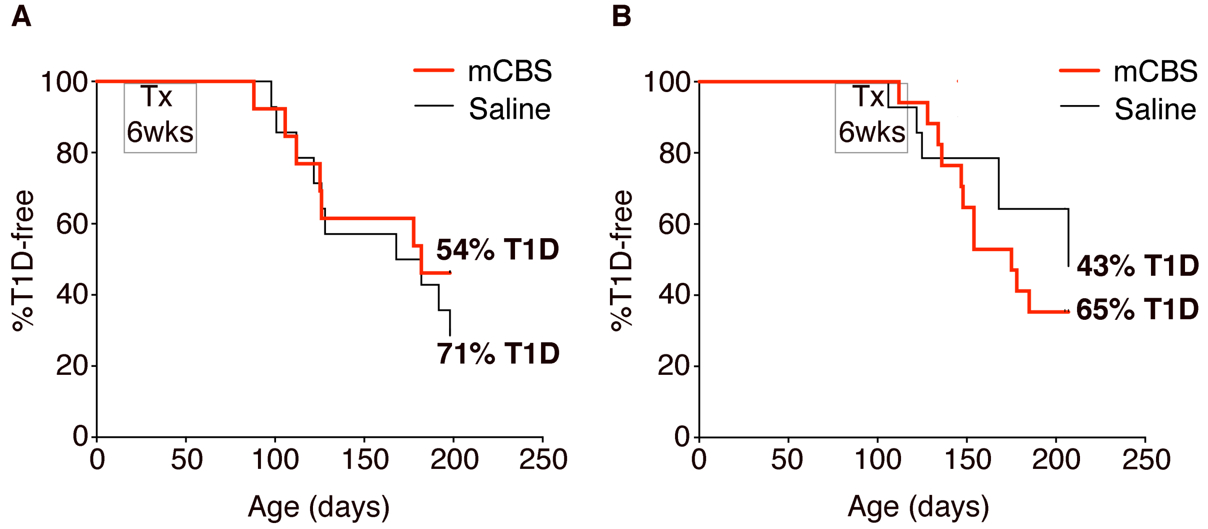

Supplemental Figure 7. T1D incidence in NOD females treated with mCBS or saline from 2 and 10-12 weeks of age. (**A**) T1D incidence was monitored in NOD females treated from 2 wks of age with mCBS (50 mg/kg/day i.p.; n=13; red) or i.p. saline (n= 14; black) for 6 weeks. (**B**) T1D incidence in 10-12 week NOD females after treatment with 50 mg/kg/day i.p. mCBS for 6 weeks (n=17; red) or saline-treated mice (n=14; black). Data is presented as percentage of mice that remained T1D-free. There were no significant differences observed between the treated groups for each age group, Fishers Exact test. Tx, treatment duration.

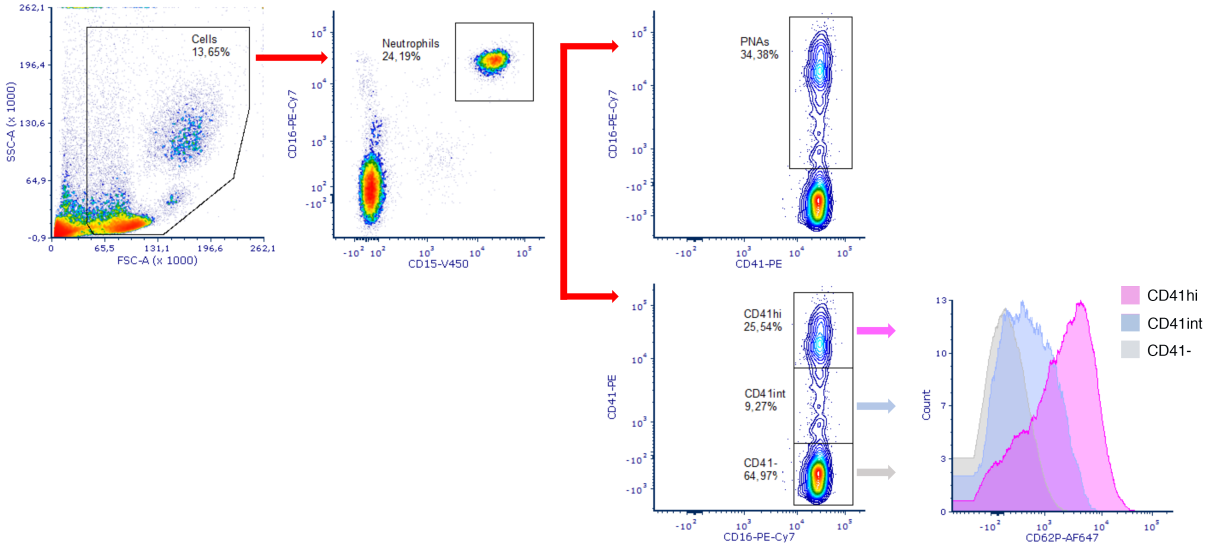

Supplemental Figure 8. Gating strategy for identifying PNAs in human peripheral blood. Neutrophils were identified as CD15+CD16+ cells in total leukocytes. The frequency of PNAs in total neutrophils is calculated as the percentage of CD41+ neutrophils. In some experiments, to assess the level of platelet activation in PNAs, the expression of the platelet activation marker CD62P was tested. A representative histogram shows CD62P expression levels on CD41- neutrophils (grey), CD41 intermediate PNAs (CD41int; blue) and CD41 high PNAs (CD41hi; pink).
